# Amino acid concentrations in serum, urine and dialysate/ultrafiltrate solutions of continuous venovenous hemodiafiltration patients

**DOI:** 10.1186/cc10971

**Published:** 2012-03-20

**Authors:** JM Lee, YJ Lee, J Hong

**Affiliations:** 1 Ajou University School of Medicine, Suwon, Kyeonggi-do, South Korea

## Introduction

A prospective study was performed for evaluating the amino acid losses during continuous venovenous hemodiafiltration (CVVHDF).

## Methods

Serum, 24-hour urine and dialysate/ultrafiltrate solutions of CVVHDF were obtained on days 1, 3, and 5 from 11 critically ill patients (five males, six females, mean age 63.0 ± 18.1 (24 to 90)) in the surgical ICU. We analyzed 40 kinds of amino acid concentrations in serum (34 samples), urine (15 samples) and dialysate/ultrafiltrate solutions (30 samples) by high-performance liquid chromatography analysis. The mean dialysate amount was 918.2 ml (600 to 1,500 ml), mean replacement fluid amount 1,136.4 ml (1,000 to 2,000 ml) and mean blood flow rate 175 ml (100 to 200 ml), respectively. Nutritional support for CVVHDF patients was guided as protein intake at 1.2 to 1.5 g/kg/day, caloric intake at 30 kcal/kg/day.

## Results

Among the analyzed 40 amino acids, the five highest mean concentration levels of 24-hour dialysate/ultrafiltrate solutions were glutamine (65,178.3 μmol/l (hereafter, all units for amino acids are μmol/l)), alanine (48,633.3), glycine (33,959.5), proline (27,701.5), lysine (26,519.4); of serum were glutamine (694.4), alanine (438.1), glycine (349.7), lysine (275.7), proline (262.4); and of 24-hour urine were glycine (1,523.0), histidine (957.5), alanine (920.7), glutamine (904.6), lysine (699.1), respectively. Amino acid concentrations of 24-hour dialysate/ultrafiltrate solutions showed significant correlation with amino acid concentrations of serum (*P *= 0.000). The mean amount of total amino acid loss on day 5 of CVVHDF was 2.8 times that of day 1 and 1.7 times that of day 3. The increase of amino acid loss according to CVVHDF progression was most prominent in glutamic acid (8.9 times from day 1 to day 5).

## Conclusion

The highest concentration level of 24-hour dialysate/ultrafiltrate solution was glutamine. The amount of amino acid loss after CVVHDF was correlated with the serum amino acid amount and increased according to CVVHDF progression.

